# Overlooking of Individuals with Cardiometabolic Risk by Evaluation of Obesity Using Waist Circumference and Body Mass Index in Middle-Aged Japanese Women

**DOI:** 10.3390/healthcare11050701

**Published:** 2023-02-27

**Authors:** Ichiro Wakabayashi

**Affiliations:** Department of Environmental and Preventive Medicine, School of Medicine, Hyogo Medical University, Nishinomiya 663-8501, Hyogo, Japan; wakabaya@hyo-med.ac.jp; Tel.: +81-798-45-6561; Fax: +81-798-45-6563

**Keywords:** body mass index, cardiometabolic risk, obesity, waist circumference, waist-to-height ratio

## Abstract

Waist circumference is often used for the diagnosis of visceral obesity and metabolic syndrome. In Japan, obesity in women is defined by the government as a waist circumference of ≥90 cm and/or BMI of ≥25 kg/m^2^. However, there has been a controversy for almost two decades as to whether waist circumference and its above-optimal cutoff are appropriate for the diagnosis of obesity in health checkups. Instead of waist circumference, the waist-to-height ratio has been recommended for the diagnosis of visceral obesity. In this study, the relationships between the waist-to-height ratio and cardiometabolic risk factors, including diabetes, hypertension and dyslipidemia, were investigated in middle-aged Japanese women (35~60 years) who were diagnosed as not having obesity according to the above Japanese criteria of obesity. The percentage of subjects showing normal waist circumference and normal BMI was 78.2%, and about one-fifth of those subjects (16.6% of the overall subjects) showed a high waist-to-height ratio. In subjects with normal waist circumference and normal BMI, odds ratios of high vs. not high waist-to-height ratio for diabetes, hypertension and dyslipidemia were significantly higher than the reference level. A considerable proportion of women who have a high cardiometabolic risk might be overlooked at annual lifestyle health checkups in Japan.

## 1. Introduction

Waist circumference is used for diagnosing metabolic syndrome in the criteria by the International Diabetes Federation [1]. In Japan, individuals aged 40~74 years are legally obliged to receive annual lifestyle health checkups for the prevention of cardiovascular diseases. In health checkups, obesity is a required item for diagnosing metabolic syndrome and is defined by the Government of Japan as a waist circumference of ≥90 cm and/or BMI of ≥25 kg/m^2^ in women [2,3]. However, there is criticism about the large optimal cutoff of waist circumference in the criteria [4,5]. In fact, in our previous study using ROC analysis for relationships between obesity indices and diabetes, the age-dependent optimal cutoffs of waist circumference for Japanese women and men aged 35~70 years were 80~82 cm and 84~85 cm, respectively [6]. In Chinese adults, the optimal cutoffs have been reported to be 75 cm in women and 85 cm in men [7]. Thus, there is a possibility that a considerable proportion of women with high cardiometabolic risk are overlooked in health checkups using the above criteria of metabolic syndrome in Japan. A previous meta-analysis study showed that the waist-to-height ratio is better than BMI and waist circumference for discriminating cardiometabolic risk [8], whereas it was shown in another meta-analysis study that there was no evidence supporting higher suitability of waist-to-height ratio than BMI and waist circumference [9]. It is not known what proportion of individuals receiving health checkups are overlooked when the above criteria of obesity using waist circumference and BMI are used for the evaluation of cardiometabolic risk in Japanese women. In this concise study, the relationships between waist-to-height ratio and cardiometabolic risk factors, including diabetes, hypertension and dyslipidemia, were therefore investigated in middle-aged women who were diagnosed as not having obesity according to the above Japanese criteria of metabolic syndrome using waist circumference and BMI.

## 2. Methods

### 2.1. Subjects

The subjects were 18,574 Japanese women aged 35~60 years who underwent annual health checkups at their workplaces. This study was approved by the Hyogo College of Medicine Ethics Committee (No. 3003 in 2020). Histories of alcohol consumption, cigarette smoking, regular exercise, medication therapy and illness were surveyed by questionnaires. In the self-written questionnaire paper, participants were first asked “Are you a habitual cigarette smoker?” Cigarette smokers were defined as participants who had smoked for 6 months or longer and had smoked for the past month or longer. Then the participants who had been smokers were further asked “What is your average cigarette consumption per day?” The response categories for this question were “20 or less cigarettes per day”, “21 or more and 40 or less cigarettes per day” and “41 or more cigarettes per day”. Because there were no subjects with very heavy cigarette consumption (41 or more cigarettes per day), the subjects were divided into three groups in this study: nonsmokers, light smokers (20 or less cigarettes per day) and heavy smokers (21 or more cigarettes per). The average alcohol consumption of each subject per week was also reported on questionnaires. Frequency of habitual alcohol drinking was asked in the questionnaire as “How frequently do you drink alcohol?” The frequency of weekly alcohol drinking was categorized as “every day” (regular drinkers), “sometimes” (occasional drinkers) and “never” (nondrinkers). Subjects with a habit of regular exercise were defined as those doing exercise almost every day for 30 min or longer per day. Thus, the subjects were divided into two groups: those with and those without a habit of regular exercise. A quantitative evaluation of regular exercise was not performed in this study.

### 2.2. Measurements

Height, body weight and waist circumference at the navel level (according to the definition of the Japanese Committee for the Diagnostic Criteria of Metabolic Syndrome [10]) were measured. Large waist circumference and high BMI were defined as ≥90 cm and ≥25 kg/m^2^, respectively. High waist-to-height ratios were defined as ≥0.51 (35~49 years of age), 0.52 (50~59 years of age) and 0.54 (60 years of age) [6]. Blood pressure was measured by trained nurses, who were part of the local health-checkup company, with a mercury sphygmomanometer once on the day of the health checkup after each subject had rested quietly in a sitting position. Korotkoff phase V was used to define diastolic pressure.

Fasted blood was collected from each subject in the morning. Serum triglycerides, HDL cholesterol and LDL cholesterol were measured by enzymatic methods using commercial kits, pureauto S TG-N, cholestest N-HDL and cholestest LDL (Sekisui Medical Co., Ltd., Tokyo, Japan), respectively. Hemoglobin A_1c_ was measured by the NGSP (National Glycohemoglobin Standardization Program)-approved technique using the latex cohesion method with a commercial kit (Determiner HbA_1c_, Kyowa Medex, Tokyo, Japan). Since the standards of hemoglobin A_1c_ used for measurement are different in the NGSP method and JDS (the Japan Diabetes Society) method, hemoglobin A_1c_ values were calibrated by using a formula proposed by JDS [11]: hemoglobin A_1c_ (NGSP) (%) = 1.02 × hemoglobin A_1c_ (JDS) (%) + 0.25%.

The criterion for each cardiometabolic risk factor was defined as follows: high blood pressure, systolic blood pressure ≥ 140 mmHg and/or diastolic blood pressure ≥ 90 mmHg [12]; low HDL cholesterol, HDL cholesterol < 50 mg/dL; high triglycerides, triglycerides ≥ 150 mg/dL [13,14]; diabetes, hemoglobin A_1c_ ≥ 6.5% [15]. Subjects receiving drug therapy for hypertension, dyslipidemia and diabetes were also included in the above definitions of hypertension, dyslipidemia and diabetes, respectively.

### 2.3. Statistical Analyses

Statistical analyses were performed using a computer software program (IBM SPSS Statistics for Windows, Version 25.0. IBM Corp, Armonk, NY, USA). In logistic regression analysis, crude and adjusted odds ratios of subjects with a high waist-to-height ratio vs. those without a high waist-to-height ratio were calculated for diabetes, hypertension or dyslipidemia. In multivariable logistic regression analysis, age, BMI, and/or habits of smoking, alcohol drinking and regular exercise were used as other explanatory variables. Categorical variables were compared by using Pearson’s chi-square test. Continuous variables were compared between the two groups with and without a high waist-to-height ratio by Student’s unpaired t-test in univariable analysis and analysis of covariance (ANCOVA) followed by Student’s t-test with Bonferroni’s multiplicity correction in multivariable analysis. Age, habits of smoking, alcohol drinking and regular exercise, and current history of medication therapy for hypertension, dyslipidemia or diabetes were adjusted in ANCOVA. Probability (*p*) values less than 0.05 were defined as significant.

## 3. Results

Table 1 shows characteristics of the overall subjects and subjects not showing large waist circumferences and high BMI. The percentages of subjects showing large waist circumference, high BMI and high waist-to-height ratio were 11.6%, 20.3% and 37.1%, respectively. The percentage of subjects showing normal waist circumference and a high waist-to-height ratio was 25.4%, while no subjects showed large waist circumference and normal waist-to-height ratio. The percentage of subjects showing normal waist circumference and normal BMI was 78.2%, and about one-fifth of those subjects (16.6% of the overall subjects) showed a high waist-to-height ratio. The mean with standard deviation and the range of waist circumferences (cm) of the subjects not showing high BMI and large waist circumference but showing high waist-to-height ratio was 83.2 ± 3.2 (71.0~89.5).

In overall subjects and subjects showing normal waist circumference and normal BMI, logistic regression analysis was performed using the waist-to-height ratio (subjects with vs. subjects without high waist-to-height ratio) as an explanatory variable and each of the cardiometabolic risk factors (diabetes, hypertension and dyslipidemia) as an independent variable (Table 2). In subjects with normal waist circumference and normal BMI, crude and adjusted odds ratios of high vs. not high waist-to-height ratio for diabetes and dyslipidemia were all significantly higher than the reference level and tended to be decreased when age and BMI were adjusted. The odds ratios for diabetes and dyslipidemia remained significant (odds ratios: 1.79 [1.28~2.51] for diabetes and 1.54 [1.40~1.70) for dyslipidemia) when age, BMI and lifestyles (histories of smoking, alcohol drinking and regular exercise) were adjusted. Crude and age- and lifestyle-adjusted odds ratios for hypertension were significantly higher than the reference level, while the odds ratios were not significantly different from the reference level when BMI was adjusted. Tendencies that are similar to the tendencies of the results in analyses using subjects who showed normal waist circumference and normal BMI were found in overall subjects, although the odds ratios tended to be higher in overall subjects than in subjects showing normal waist circumference and normal BMI.

Table 3 shows the results of comparisons by univariable analysis of cardiometabolic risk factors between the subjects without and with a high waist-to-height ratio in the subject group not showing large waist circumference and high BMI. Prevalences of diabetes, hypertension and dyslipidemia and percentages of subjects receiving medication therapy for diabetes, hypertension and dyslipidemia were significantly higher in the subjects with a high waist-to-height ratio than in the subjects without a high waist-to-height ratio. Hemoglobin A_1c_, systolic and diastolic blood pressure, log-transformed triglycerides and LDL cholesterol were significantly higher and HDL cholesterol was significantly lower in the subjects with a high waist-to-height ratio than in the subjects without a high waist-to-height ratio. These differences in cardiometabolic risk factors between the subject groups with and without high waist-to-height ratio were also found in multivariable analysis with adjustment for age, habits of smoking, alcohol drinking and regular exercise, and current history of medication therapy for diabetes, hypertension or dyslipidemia (Table 4).

## 4. Discussion

About three-fourths of the overall subjects had normal waist circumference and normal BMI levels. However, about one-fifth of those subjects had high waist-to-height ratios and showed significantly higher risks for diabetes, hypertension and dyslipidemia than those not having high waist-to-height ratios in univariable analysis and age- and lifestyle-adjusted multivariable analysis. The odds ratios for diabetes and dyslipidemia remained significant even after adjustment for BMI. Interestingly, a high waist-to-height ratio was not associated with hypertension when BMI was adjusted in the multivariable analysis. This finding is reasonable since blood pressure, which strongly depends on cardiac output, is directly influenced by general obesity but not by visceral obesity. The mean levels of the variables of cardiometabolic risk factors were also significantly different between the subject groups with and without a high waist-to-height ratio. The above findings suggest that in the group of subjects showing normal waist circumference and normal BMI levels, subjects with a high waist-to-height ratio have a higher cardiometabolic risk than subjects without a high waist-to-height ratio. This indicates that a considerable proportion (16.6% in this study) of women who have a high cardiometabolic risk were overlooked at annual lifestyle health checkups in Japan. No subjects showed a large waist circumference and normal waist-to-height ratio; in other words, all subjects with large waist circumferences showed a high waist-to-height ratio. Therefore, waist-to-height ratio, instead of waist circumference, is better for diagnosing obesity more correctly in health checkups for the purpose of early prevention of cardiovascular diseases in middle-aged women.

The above criteria of obesity using waist circumference and BMI have been used since 2008 [2] when a new law about lifestyle health checkups was established in Japan. However, there has been a controversy for almost two decades as to whether waist circumference and its above-optimal cutoff for women are appropriate for the diagnosis of obesity in health checkups [4,5]. In the present study, using the cutoff of waist circumference for women (90 cm or larger) in health checkups, the proportion of subjects diagnosed as having a large waist circumference was 11.6%, which was much lower than the proportions of subjects diagnosed as having high BMI (20.3%) and high waist-to-height ratio (37.1%). Thus, it is strongly suspected that a considerable number of individuals with visceral obesity are overlooked when the current Japanese criteria for obesity for women are used. The waist-to-height ratio is a convenient variable because the appropriate waist circumference is estimated by an easy calculation using an approximately optimal cutoff value of 0.5, i.e., the upper limit of the appropriate waist circumference (cm) is approximately half of the height (cm). A waist-to-height ratio of 0.5 or higher has been demonstrated to be a more favorable criterion for the identification of higher metabolic individuals than the criteria of other anthropometric indices including BMI, waist circumference and waist-to-hip ratio in Asians [16,17,18,19]. Age-dependent cut-off values obtained in ROC analysis with diabetes as an outcome [6] were used in this study. Using these criteria, 37.1% of overall subjects showed a high waist-to-height ratio. Further studies are needed to determine practically useful optimal cut-off values of waist-to-height ratio for predicting the risk of future cardiovascular events.

## 5. Conclusions

A considerable proportion (16.6% in this study) of women who have a high cardiometabolic risk are thought to be overlooked at annual lifestyle health checkups in Japan. Waist-to-height ratio, instead of waist circumference, is recommended to be used for the diagnosis of visceral obesity in health checkups.

## Figures and Tables

**Table 1:** Characteristics of the overall subjects and subjects not showing large waist circumference and high BMI.

	Overall	Without Large WC and High BMI
Number	18,574	14,529
Age (years)	47 9 ± 7.0	47.6 ± 7.0
Height (cm)	156.4 ± 5.6	156.6 ± 5.5
Weight (kg)	55.0 ± 9.3	51.5 ± 5.9
BMI (kg/m^2^)	22.5 ± 3.6	21.0 ± 2.1
WC (cm)	78.1 ± 9.7	74.6 ± 6.8
WHtR	0.500 ± 0.064	0.477 ± 0.046
High BMI (%)	20.3	0
Large WC (%)	11.6	0
High WHtR	37.1	21.3
Therapy for diabetes (%)	1.4	0.7
Therapy for hypertension (%)	8.4	5.8
Therapy for dyslipidemia (%)	4.5	3.2
Diabetes (%)	2.6	1.4
Hypertension (%)	20.2	14.2
Dyslipidemia (%)	36.2	23.2
Drinkers (%)		
Occasional	30.0	30.5
Regular	9.4	10.1
Smokers (%)		
Light	17.5	17.9
Heavy	0.8	0.8
Regular exercise (%)	5.3	5.4
Hemoglobin A_1c_ (%)	5.38 ± 0.52	5.32 ± 0.42
Systolic BP (mmHg)	121.9 ± 17.0	119.0 ± 15.9
Diastolic BP (mmHg)	72.3 ± 11.2	70.4 ± 10.5
Triglycerides (mg/dL)	78 (55, 115)	72 (52, 103)
HDL cholesterol (mg/dL)	65.3 ± 14.9	67.4 ± 14.8
LDL cholesterol (mg/dL)	114.0 ± 30.0	110.6 ± 29.1

Shown are numbers, proportions, means with standard deviations and medians with interquartile ranges in parentheses of the variables. WC, waist circumference; WHtR, waist-to-height ratio; BP, blood pressure.

**Table 2:** Odds ratios for diabetes, hypertension and dyslipidemia of subjects with high waist-to-height ratio vs. subjects without high waist-to-height ratio in the overall subjects and subjects not showing large waist circumference and high BMI.

	Overall (n = 18574)	Without Large WC and High BMI (n = 14529)
**Crude OR**		
Diabetes	5.56 (4.49~6.87) *	3.11 (2.35~4.14) *
Hypertension	3.46 (3.21~3.72) *	2.05 (1.85~2.27) *
Dyslipidemia	3.49 (3.27~3.71) *	2.63 (2.43~2.86) *
**Age-adjusted OR**		
Diabetes	4.82 (3.89~5.96) *	2.38 (1.78~3.17) *
Hypertension	3.04 (2.82~3.28) *	1.62 (1.45~1.80) *
Dyslipidemia	3.18 (2.98~3.39) *	2.27 (2.09~2.47) *
**Age and lifestyle-adjusted OR**		
Diabetes	4.81 (3.88~5.96) *	2.42 (1.81~3.23) *
Hypertension	3.06 (2.83~3.30) *	1.62 (1.46~1.80) *
Dyslipidemia	3.19 (2.99~3.41) *	2.30 (2.11~2.51) *
**Age, lifestyle and BMI-adjusted OR**		
Diabetes	1.97 (1.52~2.56) *	1.79 (1.28~2.51) *
Hypertension	1.02 (0.92~1.13)	1.03 (0.91~1.16)
Dyslipidemia	1.64 (1.50~1.79) *	1.54 (1.40~1.70) *

Shown are odds ratios with 95% confidence intervals in parentheses. Crude and adjusted odds ratios of subjects with high waist-to-height ratio vs. those without high waist-to-height ratio were calculated for diabetes, hypertension or dyslipidemia. In multivariable logistic regression analysis, age, BMI, and/or lifestyle (habits of smoking, alcohol drinking and regular exercise) were used as other explanatory variables. OR, odds ratio; WC, waist circumference. Asterisks denote significantly high odds ratios compared with the reference level of 1.00 (*, *p* < 0.01).

**Table 3:** Comparisons of cardiometabolic risk factors between subjects without and with a high waist-to-height ratio in the subject group not showing large waist circumference and high BMI in univariable analysis.

	Without High WHtR (n = 11,441)	With High WHtR (n = 3088)
WHtR	0.460 (0.459~0.460)	0.539 (0.538~0.540) *
Diabetes (%)	0.94	2.88 *
Hypertension (%)	12.1	22.1 *
Dyslipidemia (%)	25.0	46.8 *
Therapy for diabetes (%)	0.52	1.55 *
Therapy for hypertension (%)	12.1	22.1 *
Therapy for dyslipidemia (%)	2.67	5.08 *
Hemoglobin A_1c_ (%)	5.289 (5.282~5.296)	5.429 (5.409~5.448) *
Systolic blood pressure (mmHg)	117.8 (117.5~118.1)	123.6 (123.1~124.2) *
Diastolic blood pressure (mmHg)	69.6 (69.5~69.8)	73.1 (72.7~73.4) *
Log[triglycerides (mg/dL)]	1.849 (1.845~1.853)	1.969 (1.961~1.977) *
LDL cholesterol (mg/dL)	108.0 (107.5~108.5)	120.3 (119.2~121.4) *
HDL cholesterol (mg/dL)	68.5 (68.3~68.8)	63.0 (62.5~63.5) *

Shown are percentages and means with 95% confidence intervals of the variables. Triglycerides did not show a normal distribution and were used for analysis after logarithmic transformation with a base of 10. WHtR, waist-to-height ratio. Asterisks denote significant differences from subjects without a high waist-to-height ratio (*, *p* < 0.01).

**Table 4:** Comparisons of cardiometabolic risk factors between subjects without and with a high waist-to-height ratio in the subject group not showing large waist circumference and high BMI in multivariable analysis.

	Without High WHtR (n = 11,441)	With High WHtR (n = 3088)
Hemoglobin A_1c_ (%)	5.302 (5.295~5.309)	5.382 (5.369~5.395) *
Systolic blood pressure (mmHg)	118.3 (118.0~118.5)	121.9 (121.4~122.4) *
Diastolic blood pressure (mmHg)	69.9 (69.7~70.1)	72.1 (71.7~72.4) *
Log[triglycerides (mg/dL)]	1.853 (1.849~1.857)	1.953 (1.946~1.961) *
LDL cholesterol (mg/dL)	108.7 (108.2~109.2)	117.6 (116.6~118.5) *
HDL cholesterol (mg/dL)	68.6 (68.3~68.8)	62.8 (62.3~63.3) *

Shown are means with 95% confidence intervals of the variables. Triglycerides did not show a normal distribution and were used for analysis after logarithmic transformation with a base of 10. Age, habits of smoking, alcohol drinking and regular exercise, and current history of medication therapy for hypertension, dyslipidemia or diabetes were adjusted in ANCOVA. WHtR, waist-to-height ratio. Asterisks denote significant differences from subjects without a high waist-to-height ratio (*, *p* < 0.01).

## Data Availability

The data can be obtained by reasonable request from the corresponding author on reasonable requests.

## References

[1] Alberti K.G., Zimmet P., Shaw J., IDF Epidemiology Task Force Consensus Group (2005). The metabolic syndrome—A new worldwide definition. Lancet.

[2] Matsuzawa Y. (2005). Metabolic syndrome--definition and diagnostic criteria in Japan. J. Atheroscler. Thromb..

[3] Yamagishi K., Iso H. (2017). The criteria for metabolic syndrome and the national health screening and education system in Japan. Epidemiol. Health.

[4] Miyatake N., Wada J., Matsumoto S., Nishikawa H., Makino H., Numata T. (2007). Re-evaluation of waist circumference in metabolic syndrome: A comparison between Japanese men and women. Acta Med. Okayama.

[5] Nakamura K., Nanri H., Hara M., Higaki Y., Imaizumi T., Taguchi N., Sakamoto T., Horita M., Shinchi K., Tanaka K. (2011). Optimal cutoff values of waist circumference and the discriminatory performance of other anthropometric indices to detect the clustering of cardiovascular risk factors for metabolic syndrome in Japanese men and women. Environ. Health Prev. Med..

[6] Wakabayashi I., Daimon T. (2012). Receiver-operated characteristics (ROCs) of the relationships between obesity indices and multiple risk factors (MRFs) for atherosclerosis at different ages in men and women. Arch Gerontol. Geriatr..

[7] Zeng Q., He Y., Dong S., Zhao X., Chen Z., Song Z., Chang G., Yang F., Wang Y. (2014). Optimal cut-off values of BMI, waist circumference and waist:height ratio for defining obesity in Chinese adults. Br. J. Nutr..

[8] Ashwell M., Gunn P., Gibson S. (2012). Waist-to-height ratio is a better screening tool than waist circumference and BMI for adult cardiometabolic risk factors: Systematic review and meta-analysis. Obes. Rev..

[9] Sommer I., Teufer B., Szelag M., Nussbaumer-Streit B., Titscher V., Klerings I., Gartlehner G. (2020). The performance of anthropometric tools to determine obesity: A systematic review and meta-analysis. Sci. Rep..

[10] Japanese Society of Internal Medicine (2005). Metabolic Syndrome-Definition and Diagnostic Criteria in Japan. J. Jpn. Soc. Int. Med..

[11] Kashiwagi A., Kasuga M., Araki E., Oka Y., Hanafusa T., Ito H., Tominaga M., Oikawa S., Noda M., Kawamura T. (2012). International clinical harmonization of glycated hemoglobin in Japan: From Japan Diabetes Society to National Glycohemoglobin Standardization Program values. J. Diabetes Investig..

[12] Flack J.M., Adekola B. (2020). Blood pressure and the new ACC/AHA hypertension guidelines. Trends Cardiovasc. Med..

[13] Alberti K.G., Eckel R.H., Grundy S.M., Zimmet P.Z., Cleeman J.I., Donato K.A., Fruchart J.C., James W.P., Loria C.M., Smith S.C. (2009). Harmonizing the metabolic syndrome: A joint interim statement of the International Diabetes Federation Task Force on Epidemiology and Prevention; National Heart, Lung, and Blood Institute; American Heart Association; World Heart Federation; International Atherosclerosis Society; and International Association for the Study of Obesity. Circulation.

[14] Teramoto T., Sasaki J., Ishibashi S., Birou S., Daida H., Dohi S., Egusa G., Hiro T., Hirobe K., Iida M. (2013). Japan Atherosclerosis Society. Executive summary of the Japan Atherosclerosis Society (JAS) guidelines for the diagnosis and prevention of atherosclerotic cardiovascular diseases in Japan-2012 version. J. Atheroscler. Thromb..

[15] American Diabetes Association (2010). Diagnosis and classification of diabetes mellitus. Diabetes Care.

[16] Ho S.Y., Lam T.H., Janus E.D., Hong Kong Cardiovascular Risk Factor Prevalence Study Steering Committee (2003). Waist to stature ratio is more strongly associated with cardiovascular risk factors than other simple anthropometric indices. Ann. Epidemiol..

[17] Lyu L.C., Hsu C.Y., Yeh C.Y., Lee M.S., Huang S.H., Chen C.L. (2003). A case-control study of the association of diet and obesity with gout in Taiwan. Am. J. Clin. Nutr..

[18] Hsieh S.D., Muto T. (2005). The superiority of waist-to-height ratio as an anthropometric index to evaluate clustering of coronary risk factors among non-obese men and women. Prev. Med..

[19] Hsieh S.D., Muto T. (2006). Metabolic syndrome in Japanese men and women with special reference to the anthropometric criteria for the assessment of obesity: Proposal to use the waist-to-height ratio. Prev. Med..

